# Basal Cell Carcinoma: What You Need to Know

**Published:** 2015-05-01

**Authors:** The Editors of JADPRO

Basal cell carcinoma (BCC) is the most common cancer in the world. One out of two people will have a BCC growth (also called a lesion or tumor) before age 65. Although BCC is rarely life threatening, it should be taken seriously. If left untreated, this cancer can be disfiguring, especially on the face.

The information in these pages will help you understand more about BCC: what it is, what causes it, how it is diagnosed and treated, and how you can prevent it.

**What is basal cell carcinoma?**

Basal cell carcinoma is a type of skin cancer. It begins in the basal cells, the deepest part of the skin’s outermost layer. Basal cell carcinomas almost never spread beyond their original site to other parts of the body, especially when treated early.

**What do basal cell carcinomas look and feel like?**

As shown below, basal cell carcinomas vary widely, with a number of different appearances:

- Open sores that don’t heal
- A round ulcer that looks as though a bite has been taken out of the middle
- Red patches that have a sandpapery feel
- Shiny bumps that are raised, hard, pearly pink or gray
- An area of thickened skin
- A bump with a rolled edge
- A lesion with blood vessels that look like the spokes of a wheel

Basal cell carcinomas can occur anywhere on the body; they may appear to sit on top of the skin, or burrow into it. Most lesions are painless. Sometimes they can feel itchy. They may bleed easily if caught on clothing or nicked during shaving.

**Figure 1:**
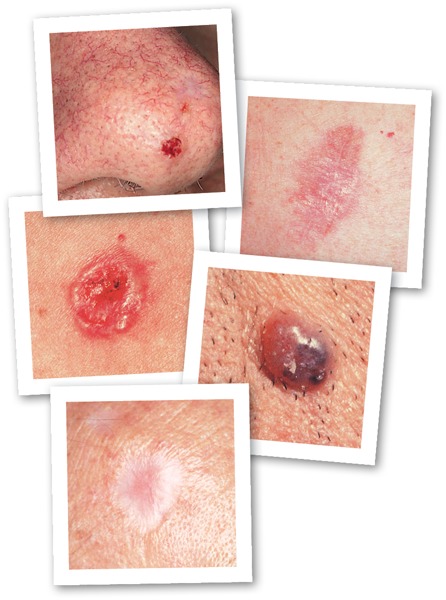
Examples of basal cell lesions. ©The Skin Cancer Foundation. SkinCancer.org. Used with permission.

**What causes basal cell carcinoma?**

The most common cause is too much exposure to ultraviolet light from the sun or tanning beds. Adults with pale skin who burn easily and who rarely tan are at the highest risk of getting BCC. People who work or play outdoors regularly also are at risk.

**How is basal cell carcinoma diagnosed?**

In most cases, a dermatologist can tell from the appearance whether a suspicious spot is BCC. If more information is required to confirm the diagnosis, he or she will perform a biopsy: removing either a small portion of the growth or the entire growth. The tissue is then examined under a microscope.

**How is basal cell carcinoma treated?**

As with most cancers, there are 3 main ways to treat BCC:

Surgery

Small lesions usually can be scraped away with a special instrument called a curette, which has a sharp, ring-shaped tip. The lesion site is then sealed with a heated needle. For larger lesions, a technique called Mohs surgery is considered the most effective way to make sure that all the cancer has been removed.

Radiation

This method is used to treat older patients and those in poor health for whom surgery would not be advisable. Tumors that cannot be easily cut out are often treated with radiation as well.

Medication

Imiquimod is used for small BCCs sitting on the surface of the skin. It is a cream that is rubbed gently into the tumor. Imiquimod works by stimulating the immune system, causing the body to produce interferon, a chemical that attacks cancer.

Fluorouracil (5-FU) is a chemotherapy drug that comes in liquid or cream form, to be rubbed into the tumor twice a day for 3 to 6 weeks. 5-FU has been associated with cure rates similar to those for imiquimod.

Vismodegib (brand name: Erivedge) is the first medicine ever approved by the US Food and Drug Administration for advanced BCC. It comes in capsule form, is taken by mouth once a day, and is used for the rare BCC that has spread throughout the body (metastasized) and cannot be treated by surgery or radiation. Due to the risk of birth defects, vismodegib should not be used by women who are pregnant or may become pregnant.

**How can I learn more about basal cell carcinoma?**

**Figure 2:**
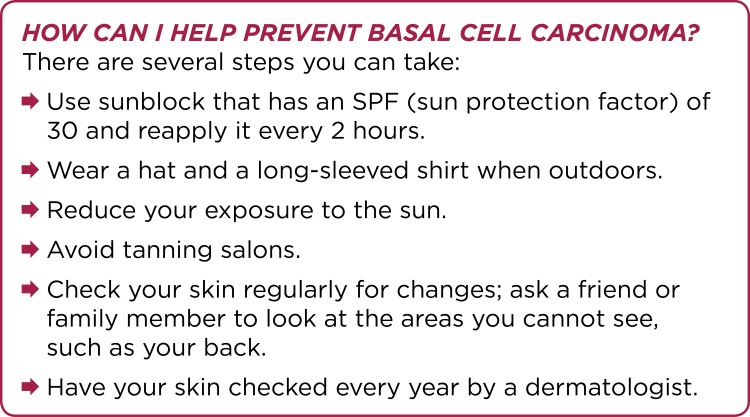
How can I help prevent basal cell carcinoma?

One excellent place to find more information about BCC is SkinCancer.org, the website of the Skin Cancer Foundation. If you use your smartphone to scan the barcode on this page, you’ll be linked to several other reliable sources of information as well.

